# Knowledge and attitude regarding pharmacogenetics among formerly pregnant women in the Netherlands and their interest in pharmacogenetic research

**DOI:** 10.1186/s12884-017-1290-z

**Published:** 2017-04-14

**Authors:** Aizati N. A. Daud, Eefke L. Bergsma, Jorieke E. H. Bergman, Hermien E. K. De Walle, Wilhelmina S. Kerstjens-Frederikse, Bert J. Bijker, Eelko Hak, Bob Wilffert

**Affiliations:** 1 Department of Pharmacy, University of Groningen, Groningen Research Institute of Pharmacy, Unit of PharmacoTherapy, -Epidemiology & -Economics, 9713AV Groningen, The Netherlands; 2 School of Pharmaceutical Sciences, Universiti Sains Malaysia, Discipline of Clinical Pharmacy, 11800 Penang, Malaysia; 3 Department of Genetics, University of Groningen, University Medical Center Groningen, 9713GZ Groningen, The Netherlands; 4 Department of Clinical Pharmacy and Pharmacology, University of Groningen, University Medical Center Groningen, 9713GZ Groningen, The Netherlands

**Keywords:** Medications, Personalized medicine, Pharmacogenetics in pregnancy, Pharmacogenetic test, Pregnancy

## Abstract

**Background:**

Pharmacogenetics is an emerging field currently being implemented to improve safety when prescribing drugs. While many women who take drugs during pregnancy would likely benefit from such personalized drug therapy, data is lacking on the awareness towards pharmacogenetics among women. We aim to determine the level of knowledge and acceptance of formerly pregnant women in the Netherlands regarding pharmacogenetics and its implementation, and their interest in pharmacogenetic research.

**Methods:**

A population-based survey using postal questionnaires was conducted among formerly pregnant women in the Northern parts of the Netherlands. A total of 986 women were invited to participate.

**Results:**

Of the 219 women who returned completed questionnaires (22.2% response rate), only 22.8% had heard of pharmacogenetics, although the majority understood the concept (64.8%). Women who had experience with drug side-effects were more likely to know about pharmacogenetics [OR = 2.06, 95% CI 1.16, 3.65]. Of the respondents, 53.9% were positive towards implementing pharmacogenetics in their future drug therapy, while 46.6% would be willing to participate in pharmacogenetic research. Among those who were either not willing or undecided in this regard, their concerns were about the consequences of the pharmacogenetic test, including the privacy and anonymity of their genetic information.

**Conclusion:**

The knowledge and attitude regarding the concept of pharmacogenetics among our population of interest is good. Also, their interest in pharmacogenetic research provides opportunities for future research related to drug use during pregnancy and fetal outcome.

**Electronic supplementary material:**

The online version of this article (doi:10.1186/s12884-017-1290-z) contains supplementary material, which is available to authorized users.

## Background

Up to eight in ten pregnant women in developed countries take prescription drugs—excluding prenatal supplements and vitamins—at some time during their pregnancy [1–3]. Pregnancy can be a crucial period for these women, as their drug efficacy and the risk of side-effects may change. Various factors influence the pharmacokinetics of drugs during pregnancy, including both physiological changes and genetic factors [4–6]. Hence, in order to personalize drug therapy for pregnant women it is imperative that this process incorporates pharmacogenetics, i.e. knowledge of the genetic factors involved in drug pharmacokinetics and drug prescribing. Such personalization is especially important when managing pharmacotherapy in pregnant women, since pre-registration trials have not yet studied the risks of drugs in this high-risk group.

Although the implementation of pharmacogenetics in drug prescribing is already underway, it is important to ensure that the public is aware and understands this new concept. In particular, they first need to allow doctors to do pharmacogenetic testing before being prescribed certain drugs that are subjected to dose changes due to patient variability. According to one survey in Denmark, the level of background knowledge on pharmacogenetics among the general public is low (14.1%) [7], although the majority of participants in other studies conducted in Australia and the USA had a positive attitude towards the concept [8–10]. However, the knowledge and attitude regarding pharmacogenetics specifically among pregnant women who also need to take their unborn child into account in drug therapy, has not yet been reported.

We therefore aimed to determine the level of knowledge of pharmacogenetics among formerly pregnant women in the Netherlands and identify the potential determinants affecting their knowledge and attitude towards this concept. Since pharmacogenetics is a relatively new field with regard to drug therapy during pregnancy, it cannot be implemented in a clinical setting until a suitable framework is in place. Such a framework requires the results of observational pharmacogenetic studies, i.e. gene-environment interaction studies, an increasing number of which are being conducted [11–14]. Therefore, we also explored the interest of formerly pregnant women to participate in future studies on pharmacogenetics.

## Methods

### Study design and settings

A population-based questionnaire was conducted between November 2015 and January 2016 to assess the knowledge and attitude of formerly pregnant women regarding pharmacogenetics. The study population included women who had been pregnant and had a history of medication use at some time in their lives. These women were identified from the University of Groningen IADB.nl pharmacy prescription database. IADB.nl contains the pharmacy data of approximately 600,000 people in several Dutch provinces, provided by 60 community pharmacies. The prescription rates of the IADB.nl population are representative of the population in the Netherlands [15]. Our study population was retrieved from a pregnancy subset of IADB.nl, based on the linkage between a child and a woman who was 15–50 years older than the child and who shared the same home address [16, 17]. Details of this linkage and of its validation have been reported previously [18].

### Participants

In November 2015, we selected participants who had given birth between 1 January 2011 and 31 December 2014. This resulted in 3689 eligible women who were registered over 37 pharmacies. Power analysis revealed that reporting of associations between the dependent variables (‘knowledge’, ‘attitude’ and ‘interest’) and determinants as independent variables would require a total of 135 respondents. This calculation was based on an odds ratio of 2, an effect size of 0.3, 80% power and 5% significance. As we estimated the response rate at 15%, we selected approximately 1000 women whom we sent the invitation letters.

We first selected community pharmacies that registered the highest number of eligible women. These pharmacies were invited to participate in the study by e-mail and/or telephone. For each participating pharmacy, we used the list of eligible women from the pregnancy subset of IADB.nl to select participants at random. We continued to invite the pharmacies until we reached 1000 eligible women. For these women, we obtained their latest address from the pharmacy and sent them a package containing an invitation letter, a questionnaire coded with a unique identification number, and a pre-paid return envelope.

### Questionnaire

The questionnaires were in Dutch and divided into five sections: a) personal information; b) experiences with diseases and drug use; c) knowledge on pharmacogenetics; d) attitude towards pharmacogenetics; and e) interest to participate in pharmacogenetic research (Additional File 1). The internal validity of the questionnaire was assessed by a methodological advisor and members of the research group, and some questions were subsequently revised to be more precise and concise. The first draft of the questionnaire was tested on a pilot group of 25 women who had a mean age of 26 years. They were sent either a paper version or an electronic version of the questionnaire, together with accompanying questions aimed at assessing their understanding of the content and its clarity; the questionnaire was then revised accordingly.

The questions were mostly closed ended, with the options of ‘Yes/No/I don’t know’. The respondents were allowed to leave blank answers. Since the concept of pharmacogenetics is new to many, we provided a description of this term in the invitation letter and in section c), after a series of questions to measure their background knowledge. The description was as follows: *‘Pharmacogenetics looks at the influence of your genetic traits on the effect of medication. It is possible that different people break down medication differently due to variations in their genetic traits’*. Although pharmacogenetics is one of the subject areas within personalized medicine, we do not introduce the broader terms (‘personalized medicine’, ‘precision medicine’, ‘individualized medicine’) in the questionnaire. It is because we focused on the relation between genetics and medication effects, and not the genetic factors related to medical diagnosis, therapy options, disease risk determination, etc.

### Statistical analysis

For statistical analysis, we grouped the level of education reported by respondents into three levels: low (primary school, lower general secondary education, and lower vocational education), middle (higher general secondary education and intermediate vocational education), and high (higher vocational education and university). The experiences with medication use and the responses to questions on attitude and willingness were reported as frequency and percentages. We used multivariable logistic regression with complete case analysis to analyze associations between potential determinants (i.e. age, educational level, experiences with medication use) and sum scores in terms of the dependent variables (i.e. knowledge of the concept of pharmacogenetics, attitude towards pharmacogenetics).

Associations were expressed as odds ratios (OR) with 95% confidence intervals. ‘Knowledge’ and ‘attitude’ were analyzed by combining the responses to three questions to give a sum score. Respondents who answered ‘Yes’ to all three questions assessing knowledge were considered to have good knowledge of the concept of pharmacogenetics. Respondents who answered ‘Yes’ to all three questions assessing their attitude were considered to have a good attitude towards the implementation of pharmacogenetics. The variable ‘interest to participate in pharmacogenetic research’ was analyzed either as “Yes” or “No/I don’t know”. Analyses were performed using PSAW Statistics, Version 22 (IBM Corporation, Armonk, NY, USA).

## Results

### Respondents’ characteristics

**Fig. 1:**
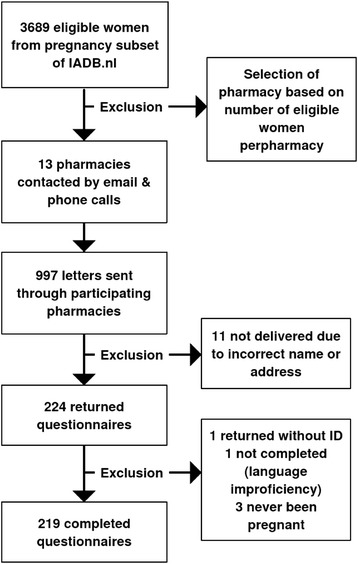
Population sampling and data collection methods

We contacted thirteen community pharmacies that had the highest number of eligible women, according to the IADB.nl database. Eight of these pharmacies agreed to participate in the study. Letters were successfully delivered to 986 selected participants, and 219 (22.2%) returned a completed questionnaire. The sampling method is depicted in Fig. 1 and the general characteristics and medical/medication history of the respondents are shown in Table 1. The mean age of the respondents was 34 years, which is older than that of all eligible women in the database (*N* = 3689, mean = 29 years, *p* <0.001). The majority of the respondents had an education level that was middle (46.1%) or high (44.3%). 22 (10%) of the respondents were pregnant at the time they filled in the questionnaire.

**Table 1:** General characteristics and medical/medication history of the respondents (*N* = 219)

Characteristics	Respondents, n (%)^a^
Age, mean years (range)	34 (25-46)
Education level^a^	
Low	18 (8.2)
Middle	101 (46.1)
High	97 (44.3)
Missing data	3 (1.4)
Living situation	
Alone/divorced	10 (4.6)
Married/living with partner/parents/others	207 (94.5)
Missing data	2 (0.9)
History of chronic disease	
Yes	55 (25.1)
Never	116 (53.0)
No, but a family member has	47 (21.5)
Missing data	1 (0.5)
History of medication use during pregnancy^b^	
Yes	94 (42.9)
No	119 (54.3)
Do not know	5 (2.3)
Missing data	1 (0.5)
History of experiencing side-effects of medication	
Yes	93 (42.5)
No	105 (47.9)
No, but a family member has	13 (5.9)
Do not know	6 (2.7)
Missing data	2 (0.9)
History of stopping medication use due to side-effects	
Yes	71 (32.4)
No	142 (64.8)
No, but a family member has	4 (1.8)
Missing data	2 (0.9)
History of stopping medication use due to inefficacy	
Yes	52 (23.7)
No	160 (73.1)
No, but a family member has	5 (2.3)
Missing data	2 (0.9)
Aware of the term ‘pharmacogenetics’	
Yes	50 (22.8)
No	166 (75.8)
Missing data	3 (1.4)
Aware of the meaning of ‘pharmacogenetics’	
Yes	38 (17.4)
No	178 (81.3)
Missing data	3 (1.4)

^a^low: primary school, lower general secondary education, and lower vocational education; middle: higher general secondary education and intermediate vocational education; high: higher vocational education and university; ^b^excluding folic acid and other supplements

Fifty five (25.1%) of the respondents had one or more chronic diseases, mostly asthma, allergy and skin conditions. Of 163 who did not have a chronic disease, 47 (28.8%) had one or more family members with at least one chronic disease. One participant did not answer this question. Nearly half of all respondents had used some medication during pregnancy, excluding folic acid and vitamin supplements (*n* = 94, 42.9%). A history of experiencing drug side-effects, not limited to the period of pregnancy, was also common among the respondents (*n* = 93, 42.5%). Furthermore, 70% of this subset of respondents admitted to stopping their medication due to side-effects. Among all respondents, 23.7% (*n* = 52) claimed that they had stopped taking medication due to ineffectiveness.

### Knowledge of pharmacogenetics

**Fig. 2:**
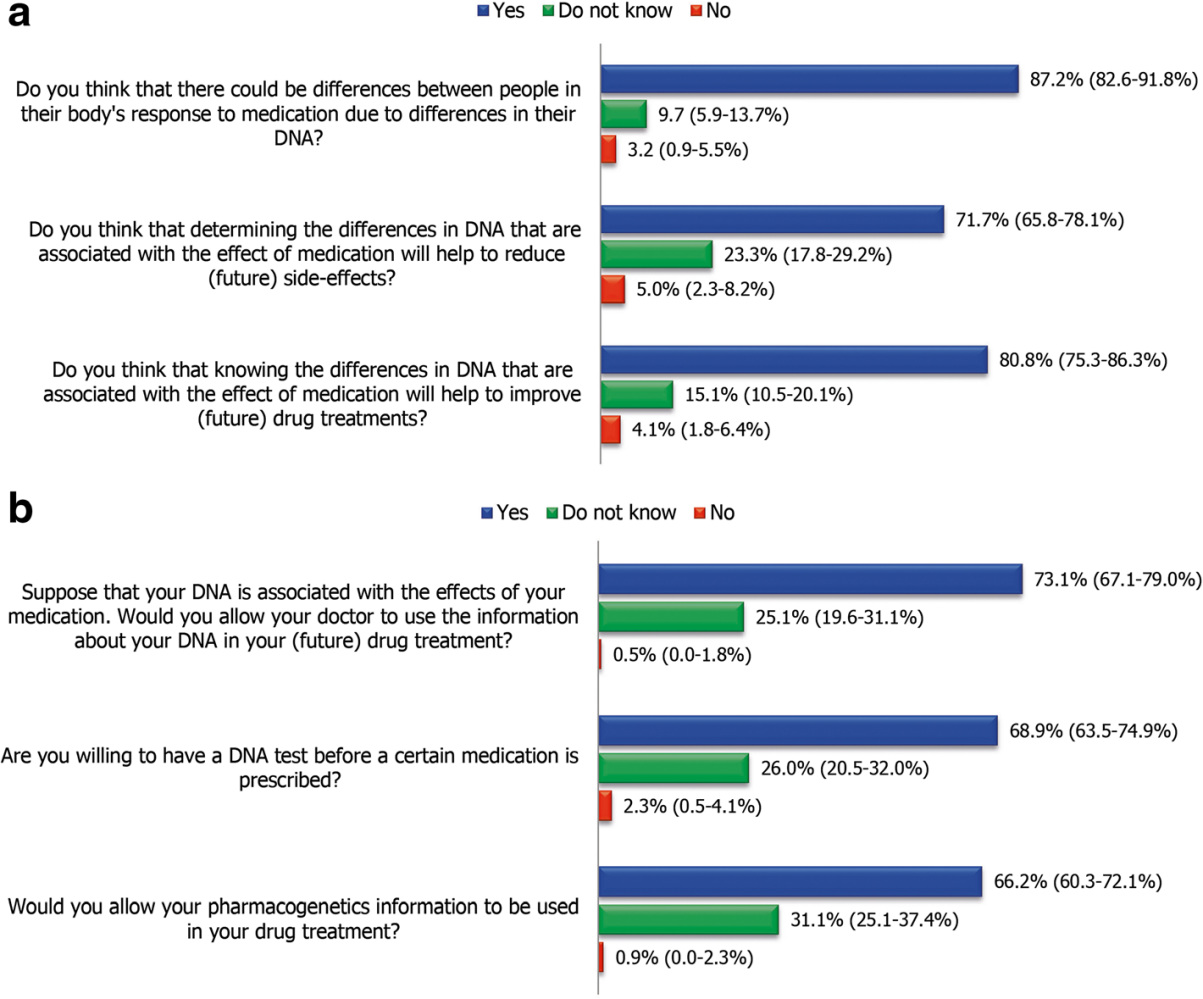
Knowledge and attitude towards pharmacogenetics among respondents (*n* = 219). **a** The responses to the questions assessing their background knowledge of the concept of pharmacogenetics; **b** The responses to the questions assessing their attitude towards the implementation of pharmacogenetics

A large percentage of the respondents were aware of the concept of pharmacogenetics (Fig. 2a). 142 (64.8%) answered positively to all three questions that assessed their background knowledge on pharmacogenetics. However, only 50 (22.8%) of the respondents had heard the term ‘pharmacogenetics’ before receiving this questionnaire, while 38 (17.4%) of them claimed that they knew its meaning. Less than 10% of the respondents had heard of a ‘DNA passport’, which is personalized identification card containing genetic information to improve drug prescribing (DNA passports are commercially available in the Netherlands). Three respondents described the DNA passport correctly, while nine described it as containing an individual’s genetic information in a broader scope, without mentioning the relation to drug use. Only one respondent had had a pharmacogenetic test, while five others knew someone who had had such a test.

Respondents with a high or medium level of education were more likely to agree with the statement *“Do you think that there could be differences between people in their body's response to medication due to differences in their DNA?”*, than were respondents with a low level of education ([OR for middle level of education = 4.84, 95% CI 1.56, 15.0]; [OR for high level = 8.10, 95% CI 2.39, 27.51]) (Table 2). Respondents who had experience with drug side-effects, either themselves or their family members, were twice as likely to have a good knowledge of the pharmacogenetics concept [OR = 2.06, 95% CI 1.16, 3.65]. Self-reported awareness of the term ‘pharmacogenetics’ and of the meaning of this term were not, however, associated with a good knowledge of pharmacogenetics.

**Table 2:** The likelihood of having good knowledge about the concept of pharmacogenetics given respondent characteristics

Characteristics	Gave positive answers to knowledge questions, adjusted OR (95% CI)^a^, *p* value			
	Do you think that there could be differences between people in their body's response to medication due to differences in their DNA?	Do you think that determining the differences in DNA that are associated with the effect of medication will help to reduce (future) side-effects?	Do you think that knowing the differences in DNA that are associated with the effect of medication will help to improve (future) drug treatments?	Answering ‘Yes’ to all three questions (sum score of 3)
Educational level				
Low	Reference level			
Middle	**4.84 (1.56-15.0), 0.006**	1.08 (0.37-3.18), 0.88	1.34 (0.43-4.16), 0.62	1.62 (0.59-4.45), 0.35
High	**8.1 (2.39-27.51), 0.001**	1.65 (0.55-4.96), 0.37	2.69 (0.81-8.91), 0.11	2.57 (0.92-7.18), 0.07
Living situation (Living with spouse/partner/others vs. alone/divorced)	0.74 (0.09-6.07), 0.78	0.56 (0.12-2.76), 0.56	1.02 (0.21-5.02), 0.98	0.75 (0.19-3.0), 0.75
Having chronic disease(s)^b^ (Yes vs. No)	0.91 (0.36-2.32), 0.85	1.11 (0.55-2.23), 0.77	1.04 (0.47-2.32), 0.92	1.41 (0.72-2.875), 0.32
Having chronic disease(s)^c^ (Yes vs. No)	0.88 (0.38-2.0), 0.75	1.47 (0.80-2.71), 0.21	1.27 (0.63-2.56), 0.50	1.70 (0.96-3.02), 0.071
Used medication during pregnancy (Yes vs. No/Do not know)	0.99 (0.44-2.26), 0.99	1.62 (0.88-2.96), 0.12	1.40 (0.70-2.79), 0.34	1.23 (0.70-2.18), 0.47
Experienced side effect^b^ (Yes vs. No/Do not know)	1.97 (0.82-4.74), 0.13	1.45 (0.79-2.67), 0.23	**2.13 (1.02-4.45), 0.045**	1.65 (0.93-2.94), 0.09
Experienced side effect^c^ (Yes vs. No/Do not know)	**2.66 (1.11-6.40), 0.029**	1.74 (0.95-3.20), 0.073	**2.56 (1.24-5.29), 0.011**	**2.06 (1.16-3.65), 0.014**
Stopping medication due to side effect(s) (Yes vs. No/do not know)	0.98 (0.42-2.32), 0.97	1.30 (0.68-2.48), 0.43	1.08 (0.52-2.24), 0.84	1.47 (0.80-2.71), 0.22
Stopping medication due to inefficacy (Yes vs. No/Do not know)	1.10 (0.42-2.90), 0.85	1.06 (0.52-2.14), 0.88	0.60 (0.28-1.27), 0.18	0.97 (0.50-1.88), 0.93
Aware of the term ‘pharmacogenetics’	4.08 (0.93-17.91), 0.063	1.37 (0.64-2.93), 0.42	1.79 (0.70-4.58), 0.22	1.57 (0.77-3.19), 0.22
Aware of the meaning of ‘pharmacogenetics’	2.88 (0.65-12.75), 0.16	1.53 (0.65-3.60), 0.33	3.01 (0.87-10.36), 0.081	1.80 (0.80-4.07), 0.16

^a^adjusted for age; ^b^themselves; ^c^themselves or family members; bold font indicates significant associations

### Attitude towards pharmacogenetics

More than half of the respondents had a positive attitude towards pharmacogenetics (Fig. 2b). 118 (53.9%) answered positively to all three questions assessing their attitude towards the implementation of pharmacogenetics in any drug treatments that they may need in the future. 20–30% of the respondents were unsure of whether they would take a DNA test or would allow their doctors to use their pharmacogenetic information in their drug treatment. The most preferred method of DNA collection, voted as the first choice, was buccal swab collection (57.8%), followed by saliva collection (25.4%), dried blood spot (15%), and the least preferred method being taking a blood sample (1.2%) (Additional file 2: Figure S1). A positive attitude towards the implementation of pharmacogenetics was associated with having good knowledge of pharmacogenetics [OR = 3.50, 95% CI 1.95, 6.30]. Other variables were not found to be significantly correlated with a positive attitude towards pharmacogenetics (Additional file 1: Table S1).

### Interest to participate in pharmacogenetic research

We determined respondents’ interest to take part in future pharmacogenetic research: 102 (46.6%) were positive, 31 (14.2%) answered ‘No’, while 85 (38.8%) answered ‘Do not know’. Among those who responded ‘No’ or ‘Do not know’ (*n* = 116), the reason most often given for not wanting to participate in such research was that they were worried about the consequences (35.3%), while some of them did not want their DNA or genetic information to be used for research (12.9%). Many of them also described their concerns in the open-ended option provided and some of these are listed in Table 3. In addition, nearly half of the respondents were interested in obtaining more information on pharmacogenetics (*n* = 98, 44.7%), while the percentages of those who declined or who were undecided were almost equal (23.7% and 29.2%, respectively). The preferred source of information was the internet (*n* = 103, 47.0%), followed by an information leaflet (*n* = 91, 41.6%), advice from their general practitioner (*n* = 90, 41.1%), and advice from a pharmacist (*n* = 41, 18.7%).

**Table 3:** Reasons not to participate in pharmacogenetic research (*N* = 116)

Reasons	Number	Percent^a^
‘I am worried about the consequences’	41	35.3
‘I do not want my DNA/genetic information to be used in research’	15	12.9
‘I am not interested in pharmacogenetic research’	10	8.6
‘I do not understand the benefit of genetic testing’	8	6.9
Others (answers in open-ended option):		
‘Need more information about the research before I can decide’	2	19.0
‘Concerned about the privacy/anonymity of my genetic information and afraid it will be used/abused by insurance company, employer, etc.’	14	12.1
‘Need more time to think about this’	5	4.3
‘Lack of understanding or doubtful about this concept’	3	2.6
‘Do not like/no time to participate in research’	2	1.7
Other/personal reasons	5	4.3

^a^percentages may add up to more than 100 because respondents could give more than one answer

Good knowledge of the concept of pharmacogenetics and a positive attitude towards its implementation were significantly associated with the interest to participate in pharmacogenetic research [OR for ‘knowledge’ = 2.05, 95% CI 1.15, 3.66; OR for ‘attitude’ = 5.73, 95% CI 3.16, 10.37]. Women who had good knowledge and a positive attitude were also more likely to be interested in obtaining more information about the concept [OR for ‘knowledge’ = 1.85, 95% CI 1.03, 3.32; OR for ‘attitude’ = 2.25, 95% CI 1.29, 3.92].

## Discussion

This study suggests that while most women who have been pregnant are not familiar with the term ‘pharmacogenetics’, many do have some understanding of the association between DNA and drug therapy effects. Our results also show that while more than half of formerly pregnant women are positive towards the use of their genetic information in determining their future drug therapy, many of them are either neutral or still undecided.

### Predictors of good knowledge and attitude towards pharmacogenetics

We found educational level to be one of the predictors of adequate knowledge on pharmacogenetics, while the majority of the respondents had at least a medium level of education. The high percentage of highly educated women among our respondents (44.3%) is similar to the percentage reported by Statistics Netherlands in 2016 for women aged between 25 and 34 years (48%) [19]. The percentage of respondents who reported they had used medication during pregnancy (42.9%) is lower than the percentage of the source population who had received any prescription during pregnancy (71.4%, *N* = 3689), and also lower than the percentage reported previously in the same population (69.2%) [16]. We expect that the actual drug use during pregnancy in our respondents was higher, since our data is based on self-reported events and subject to recall bias.

Our finding that respondents who had experienced drug side-effects themselves—or who had family members with side-effects—were more likely to have a good understanding and knowledge of the pharmacogenetics concept is consistent with the findings of Nielsen and colleagues [7]. Others have also found a history of side-effects to be a good motivation for pharmacogenetic testing [10, 20], even if the individual is aware of the possibility of the DNA sample being accessed without the patient’s permission or if the sampling requires a blood test [10].

Women in our study population were generally positive and optimistic regarding the use of pharmacogenetics information for their future drug therapy. This attitude towards pharmacogenetics was not influenced by education level, chronic disease or a history of side-effects, but was significantly associated with a good understanding of the concept. The fact that when answering questions about their attitudes many respondents indicated they were undecided or unsure—rather than giving a negative answer—is not surprising. After all, a large majority of them might only have heard of pharmacogenetics for the first time from the questionnaire in this study, which may well explain their conservative opinions. Other reasons that might explain the reluctance in accepting pharmacogenetics are the lack of understanding or belief that this concept could help in their drug treatment, or they might be worried about the consequences [20, 21].

Our results suggest that neither a history of chronic diseases nor medication use during pregnancy leads to a more positive attitude or perception on pharmacogenetics. In addition, the number of medications consumed also did not determine the attitude towards pharmacogenetics among the public [7]. This finding shows that the concept of pharmacogenetics can be universally accepted by the public, regardless of health status and disease burden; we predict the public may well accept the use of pharmacogenetics as long as they are well informed of its possibilities.

### Interest in pharmacogenetic research

In our study, the respondents’ preferred sources of information about pharmacogenetics were different to those found in other studies. While we found the internet and an information leaflet to be most favored, others report the advice from doctors, a specialist or other healthcare providers to be more popular [20, 22]. One of the possible explanations is that we provided a link to a reliable website on pharmacogenetics in the questionnaire, which might aid their decision to choose the internet. Regardless of their choice, our questionnaire itself might well stimulate participants’ interest in finding out more about pharmacogenetics. Indeed, better knowledge on the concept of pharmacogenetics, and a positive attitude towards the implementation of pharmacogenetics were associated with a high interest to participate in future pharmacogenetic research. Concerns reported here such as those regarding the privacy and anonymity of genetic information —and possible misuse by employers or insurance companies—have also been reported by others [8, 20, 23, 24]. This emphasizes the need for regulatory measures to be established to protect patients’ privacy.

There are three main limitations to our study. First, sampling bias may have arisen during the recruitment of community pharmacies, since we could only select those pharmacies registered in the pregnancy subset of IADB.nl, and several pharmacists declined to participate because the topic was deemed too difficult for their population. Second, information bias may also have affected our results in terms of respondents’ knowledge about pharmacogenetics. Both the cover letter and the questionnaire provided respondents with a brief explanation of pharmacogenetics. While this information was intended to introduce this concept to those who were not familiar with it, and to promote interest in filling in the questionnaire [22, 23], it may have assisted respondents in answering the questions, and resulted in a higher level of knowledge being reported. Third, there was a selection bias towards women who were more health-literate, and away from those who chose not to respond to the questionnaire. Although our survey results may not represent the entire Dutch female population, they do focus on a population who might be pregnant, and provide a useful picture of their views on pharmacogenetics and its implementation in their drug therapy.

A key strength of our study is that we are the first to report on the knowledge and attitude regarding pharmacogenetics specifically in a formerly pregnant population. While awareness of pharmacogenetics itself appears to be low, women’s knowledge and attitude on the concept is good. Their acceptance and concerns regarding pharmacogenetic issues do not appear to be very different from those among the public and patient population as reported elsewhere [7, 8, 10, 20, 22]. Future research in this area could include longitudinal studies of pregnant women and also exploring attitudes to DNA testing for pharmacogenetic information, before, during and after pregnancy.

## Conclusion

The substantial level of interest in pharmacogenetics among formerly pregnant women is encouraging; therefore it is worthwhile pursuing its application in personalized drug therapy for safer use of drugs during pregnancy. Several designs in genetic epidemiology research may pave ways to understand the role of pharmacogenetics in fetal drug exposure and outcome. A question remained unanswered is whether the privacy and confidentiality of the genetic information can be adequately assured, in both research and clinical settings, which need to be addressed by researchers and health authorities.

## Additional files

Additional file 1: Questionnaire used in the survey (translated in English). **Table S1:** The likelihood of having a good attitude towards pharmacogenetics testing given respondent characteristics (PDF 510 kb)

Additional file 2: **Figure S1.** Preferred DNA collection methods among respondents (*n* = 173). (TIF 312 kb)

## Abbreviation

OR: Odds ratio
