# Supplementary material for: Knowledge and attitude regarding pharmacogenetics among formerly pregnant women in the Netherlands and their interest in pharmacogenetic research
Source: BMC Pregnancy Childbirth. 2017 Apr 14;17:120. doi: 10.1186/s12884-017-1290-z (PMC5391584; doi:10.1186/s12884-017-1290-z)
Supplement: Supplementary file 1 — Questionnaire used in the survey (translated in English). Table S1: The likelihood of having a good attitude towards pharmacogenetics testing given respondent characteristics (PDF 510 kb) [file 12884_2017_1290_MOESM1_ESM.pdf]

Knowledge and attitude regarding pharmacogenetics among formerly pregnant women in the Netherlands and their interest in pharmacogenetic research

Aizati N.A. Daud†, *University of Groningen, Groningen Research Institute of Pharmacy, Unit of PharmacoTherapy, -Epidemiology & -Economics, Dept. of Pharmacy, 9713AV Groningen, the Netherlands; Universiti Sains Malaysia, Discipline of Clinical Pharmacy, School of Pharmaceutical Sciences, 11800 Penang, Malaysia;* n.a.a.daud@rug.nl

Eefke L. Bergsma, *University of Groningen, Groningen Research Institute of Pharmacy, Unit of PharmacoTherapy, -Epidemiology & -Economics, Dept. of Pharmacy, 9713AV Groningen, the Netherlands;* e.l.bergsma@student.rug.nl

Jorieke E.H. Bergman, *University of Groningen, University Medical Center Groningen, Dept. of Genetics, 9713GZ Groningen, the Netherlands;* j.e.h.van.kammen@umcg.nl

Hermien E.K. De Walle, *University of Groningen, University Medical Center Groningen, Dept. of Genetics, 9713GZ Groningen, the Netherlands;* h.e.k.de.walle@umcg.nl

Wilhelmina S. Kerstjens-Frederikse, *University of Groningen, University Medical Center Groningen, Dept. of Genetics, 9713GZ Groningen, the Netherlands;* w.s.kerstjens@umcg.nl

Bert J. Bijker, *University of Groningen, Unit of PharmacoTherapy, -Epidemiology & -Economics, Dept. of Pharmacy, 9713AV Groningen, the Netherlands;* b.j.bijker@rug.nl

Eelko Hak, *University of Groningen, Groningen Research Institute of Pharmacy, Unit of PharmacoTherapy, -Epidemiology & -Economics, Dept. of Pharmacy, 9713AV Groningen, the Netherlands;* e.hak@rug.nl

Bob Wilffert, *University of Groningen, Groningen Research Institute of Pharmacy, Unit of PharmacoTherapy, -Epidemiology & -Economics, Dept. of Pharmacy, 9713AV Groningen, the Netherlands; University of Groningen, University Medical Center Groningen, Dept. of Clinical Pharmacy and Pharmacology, 9713GZ Groningen, the Netherlands;* b.wilffert@rug.nl

†Corresponding author

Additional File 1: Questionnaire used in the survey (translated in English)

This questionnaire has 5 sections containing questions about: A) personal information, B) your experiences with diseases and medication use, C) your knowledge on pharmacogenetics, D) your attitude towards pharmacogenetics, and E) your interest to participate in pharmacogenetic research.

The participation in this survey is voluntary. If you do not want to answer a particular question, you are of course free to do so.

Please write the answers to open questions in the space provided. If you need more space, please use the back of the paper and indicate the question number.

For all other questions, please use the checkboxes. If you want to change your answer, please put a line through the checked answer and then choose the appropriate box. Always check only one answer except if stated otherwise.

Personal information

It is important to find out several information about you, so that we may better the differences in the results.

1. What is your age?
   …………

2. Are you pregnant at this moment?
   ☐Yes, namely ……….. weeks.
   ☐No
   ☐I do not know

3. Have you ever been pregnant?
   ☐Yes
   ☐No
   ☐I do not know

4. What is your highest level of completed education?
   ☐Lagere school, elementary school
   ☐Lbo (lager beroepsonderwijs), vbo, lts, lhno, vmbo
   ☐Mavo, vmbo-t, mbo-kort
   ☐Mbo, mts, meao
   ☐Havo, vwo, gymnasium
   ☐Hbo
   ☐University (bachelor or master)
   ☐Otherwise, namely:…………………………………………………………………………

5. What is your living situation?
   ☐Single
   ☐Married
   ☐Divorced/widowed
   ☐Living together with a partner
   ☐Living with my parents

☐Otherwise, namely: ……………………………………………………………………………………

Experiences

Below are some questions about your personal experiences with diseases and medication use.

6. Do you have one or several chronic disease(s) (e.g. diabetes or asthma)? If yes, which one (name them all)?
   ☐Yes, namely: ....
   ☐No
   ☐No, but a family member has

7. If you have ever been pregnant: Have you used medication during one of your pregnancies, at any time during the pregnancy, with the exception of supplements (e.g. folic acid or other vitamins)?
   ☐Not applicable, I have never been pregnant
   ☐Yes
   ☐No
   ☐I do not know anymore

8. Have you ever experienced a side effect of a medication?
   ☐Yes
   ☐No
   ☐No, but a family member has
   ☐I do not know

9. Have you ever stopped taking a medication because of the side effects of this medicine?
   ☐Yes
   ☐No
   ☐I do not know

10. Have you ever stopped taking a medication because you felt that it did not work?
    ☐Yes
    ☐No
    ☐I do not know

Knowledge

These questions enable us to know about your understanding of pharmacogenetics. If you do not know or are not sure, you can also indicate this as the answer.

The DNA stores your hereditary characteristics.

11. Do you think that there could be differences between people in their body's response to medication due to differences in their DNA?
    ☐Yes
    ☐No
    ☐I do not know

12. Do you think that determining the differences in DNA that are associated with the effect of medication will help to reduce (future) side effects?
☐Yes
☐No
☐I do not know

13. Do you think that knowing the differences in DNA that are associated with the effect of medication will help to improve (future) drug treatments?
☐Yes
☐No
☐I do not know

'Pharmacogenetics looks at the influence of your genetic traits on the effect of medication. It is possible that different people break down medication differently due to variations in their genetic traits'.

14. Before you received this questionnaire, have you ever heard of the term 'pharmacogenetics'?
☐Yes
☐No

15. Before you received this questionnaire, did you know the meaning of 'pharmacogenetics'?
☐Yes
☐No

16. Have you ever had a pharmacogenetic test? In a pharmacogenetic test, your hereditary traits related to the effect of a medication will be examined.
☐Yes
☐No
☐No, but I have had another kind of DNA-test
☐I do not know

17. Do you know someone who has had a pharmacogenetic test?
☐Yes
☐No
☐No, but I know someone who has had another type of DNA-test

18. Have you ever heard of a 'DNA-passport for pharmacogenetics'?
☐Yes
☐No, go to question 20

19. Could you describe, based on your own understanding, the meaning of a 'DNA-passport for pharmacogenetics'?
……………………………………………………………………………………………………
……………………………………………………………………………………………………
………………………………………………………………
☐I have no idea

Attitude

Below are several questions about your views and attitude towards pharmacogenetics.

20. Suppose that your DNA is associated with the effects of your medication. Would you allow your doctor to use the information about your DNA in your (future) drug treatment?
    ☐Yes
    ☐No
    ☐I do not know, I have never thought about this
    ☐I have been thinking about this, but I do not know yet

21. Are you willing to have a DNA-test before a certain medication is prescribed?
    ☐ Yes, go to question 22
    ☐ No, go to question 23
    ☐ I do not know, go to question 22

22. Which method of collecting DNA do you prefer? You can sort the options from the first preference until the fourth. You can also specify it when you think an option is unacceptable.

	1st	2nd	3rd	4th	Unacceptable
Saliva collection in a little pot	☐	☐	☐	☐	☐
Taking a cheek swab with a cotton swab	☐	☐	☐	☐	☐
Prick in the finger to collect a droplet of blood (e.g. as used by people with diabetes to check blood glucose)	☐	☐	☐	☐	☐
Blood collection by injection needle	☐	☐	☐	☐	☐

23. Do you want your pharmacogenetic information to be used in your treatment with medicines?
    ☐Yes, during the whole treatment
    ☐Yes, but only during my (potential) pregnancy
    ☐No
    ☐No opinion/I do not know

Willingness
The following questions are about your willingness to participate in future research in this area.

24. Would you take part in future research related to pharmacogenetics?
    ☐Yes, go to question 26
    ☐No, go to question 25
    ☐I do not know, go to question 25

25. If not, or in doubt, what is the reason (multiple answers are correct)?
    ☐I do not allow my DNA/genetic information to be used in research
    ☐I do not understand the benefit of genetic testing

☐I am worried about the consequences
☐I am not interested in pharmacogenetic research
☐Otherwise, please describe: ....

26. Would you like to know more about DNA-tests for pharmacogenetics?
    ☐Yes
    ☐No
    ☐I do not know

27. Which method of getting information about DNA-tests for pharmacogenetics do you prefer (multiple answers are correct)?
    ☐Internet. If you are interested, go to this link: www.farmacogenetica.nl
    ☐Information leaflet
    ☐Advice from your pharmacist
    ☐Advice from your GP
    ☐Otherwise, please describe:
    ……………………………………………………………………………………...

Thank you for completing this survey. Please return this survey with the enclosed prepaid return envelope at your earliest convenience.
If you have any questions about this questionnaire or about this research in general, please send an email to: xxxxx or call xxxx.

Additional Table 1: The likelihood of having a good attitude towards pharmacogenetics testing given respondent characteristics

Characteristics	Gave positive answers to attitude questions, adjusted OR (95% CI) *, p value			
	Allows doctors to use DNA information in (future) drug treatment	Willing to get a DNA-test before a certain medication is prescribed	Allows pharmacogenetic information to be used in (future) drug treatment	Sum score 3 to attitude questions
**Educational level**				
**Low**	Reference level			
**Middle**	0.94 (0.28-3.15), 0.92	1.01 (0.33-3.12), 0.98	1.34 (0.47-3.80), 0.58	1.41 (0.51-3.88), 0.59
**High**	0.69 (0.21-2.29), 0.55	0.86 (0.28-2.63), 0.79	1.34 (0.47-3.80), 0.58	1.61 (0.58-4.43), 0.36
**Living situation (Living with spouse/partner/ others vs. alone/ divorced)**	0.30 (0.037-2.40), 0.2	0.58 (0.12-2.80), 0.49	0.87 (0.22-3.46), 0.89	0.47 (0.12-1.89), 0.29
**Having chronic disease(s) † (Yes vs. No)**	1.37 (0.66-2.85), 0.40	1.66 (0.80-3.44), 0.18	1.25 (0.64-2.45), 0.52	1.27 (0.68-2.38), 0.46
**Having chronic disease(s)†† (Yes vs. No)**	1.28 (0.69-2.38), 0.43	1.36 (0.74-2.48), 0.32	1.57 (0.88-2.82), 0.13	1.39 (0.81-2.39), 0.24
**Used medication during pregnancy (Yes vs. No/Do not know)**	0.71 (0.38-1.32), 0.28	1.27 (0.69-2.33), 0.45	1.17 (0.65-2.10), 0.60	0.86 (0.50-1.48), 0.58
**Experienced side effect (Yes vs. No/Do not know)**	0.86 (0.46-1.59), 0.63	1.57 (0.85-2.91), 0.15	1.51 (0.83-2.72), 0.18	1.24 (0.72-2.14), 0.44
**Experienced side effect†† (Yes vs. No/Do not know)**	0.90 (0.48-1.66), 0.73	1.25 (0.69-2.26), 0.47	1.40 (0.78-2.50), 0.26	1.0 (0.58-1.70), 0.97
**Stopping medication due to side effect(s) (Yes vs. No/Do not know)**	1.03 (0.53-1.98), 0.93	1.50 (0.78-2.87), 0.23	1.66 (0.88-3.13), 0.12	1.18 (0.67-2.10), 0.56
**Stopping medication due to inefficacy (Yes vs. No/Do not know)**	1.11 (0.53-2.32), 0.78	0.69 (0.34-1.36), 0.28	0.89 (0.45-1.74), 0.73	0.85 (0.45-1.60), 0.62
**Aware of the term ‘pharmacogenetics’**	1.52 (0.70-3.30), 0.29	1.2 (0.58-2.47), 0.62	1.66 (0.80-3.43), 0.18	1.17 (0.61-2.23), 0.64
**Aware of the meaning of ‘pharmacogenetics’**	1.65 (0.68-4.01), 0.27	1.38 (0.61-3.12), 0.44	1.34 (0.61-2.97), 0.47	1.15 (0.56-2.34) 0.71
**Knowledge about pharmacogenetics (sum score of 3 to knowledge questions vs. others)**	**2.68 (1.43-5.04), 0.002**	**2.78 (1.50-5.14), 0.001**	**4.75 (2.56-8.81), <0.001**	**3.43 (1.90-6.17), <0.001**

adjusted for age; †themselves; ††themselves or family members
