# Supplementary figures and images for: Knowledge and attitude regarding pharmacogenetics among formerly pregnant women in the Netherlands and their interest in pharmacogenetic research
Source: BMC Pregnancy Childbirth. 2017 Apr 14;17:120. doi: 10.1186/s12884-017-1290-z (PMC5391584; doi:10.1186/s12884-017-1290-z)

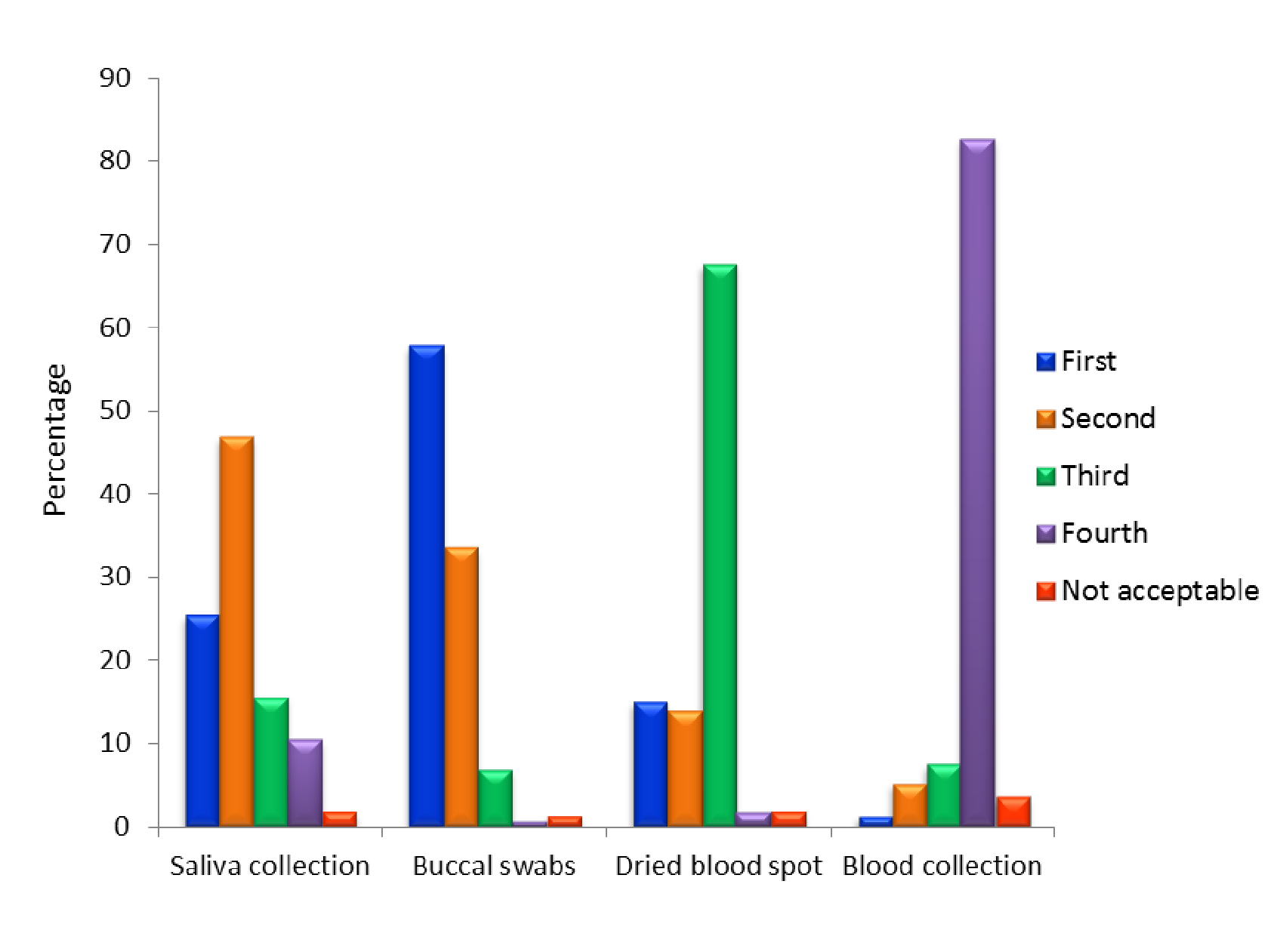

Supplement: Supplementary file 2 — Figure S1. Preferred DNA collection methods among respondents (n = 173). (TIF 312 kb) [file 12884_2017_1290_MOESM2_ESM.tif]
